# Development of a biodegradable and eco-friendly novel printing composite using biomaterials on textile substrate and assessing the characterization

**DOI:** 10.1038/s41598-025-99507-6

**Published:** 2025-05-06

**Authors:** Abdullah Al Tahsin, Tonmoy Saha, Satu Saha, Sukarna Saha, Faishal Ahamed

**Affiliations:** 1 Department of Textile Engineering, National Institute of Textile Engineering and Research (NITER), University of Dhaka, Dhaka, Bangladesh; 2 Department of Bioproducts and Biosystems, School of Chemical Engineering, Aalto University, 02150 Espoo, Finland

**Keywords:** Textile printing, Chitosan, Ultrasonication, Sustainable printing, Color fastness, Environmental sciences, Chemistry, Engineering, Materials science

## Abstract

The use of biomaterials has grown in interest over the past few years for their unique properties and diverse applications. The textile printing industry is one of the appropriate sectors to introduce biomaterials that can replace synthetic printing ingredients and reduce environmental threats. In this research, a novel textile printing process was developed using a combination of eco-friendly printing ingredients and later applied with screen printing technology on different textile substrates such as cotton, poly-cotton (65% polyester and 35% cotton), and linen. To evaluate the performance of different printed fabrics, spectrophotometric and color fastness properties were assessed and compared against the conventionally printed control samples. The CIE L*a*b* values showed a slight difference in color intensity between conventional and sustainable printing. For non-washed samples, a prominent fastness result of the highest rating was observed for sustainable-printed specimens. The results were also consistent with the number of washings, as 10 times washed samples also showed comparatively better results in the fastness property and color staining. This eco-friendly printing can replace detrimental synthetic ingredients without compromising the environment, performance, or effectiveness and sustainable printing is a potentially feasible and suitable alternative to conventional printing.

**Supplementary Information:**

The online version contains supplementary material available at 10.1038/s41598-025-99507-6.

## Introduction

Printing is a crucial process in the coloration of fabrics which has a numerous application and areas to study. Pigment printing is generally a printing process wherein the insoluble pigments with no affinity for fiber are fastened onto the fabric using binding agents in the desired configuration^1^. Because of the quick production time and ease of operation, pigment accounts for around 50% of printed textiles globally^2–5^. The traditional printing process used in the industries discharges large amounts of water and toxic chemicals like Formaldehyde, Urea, Melamine, and so on^6^. The study by Xie et al., 2022 showed the environmental impact due to conventional printing risks the wastage of energy, emission, and natural resources^7^. The International Agency for Research on Cancer (IARC) categorized formaldehyde as a Group 1 carcinogen^8^. A comprehensive mitigation strategy is necessary to address the high reactivity, volatility, and toxicity of formaldehyde. To resolve these issues, researchers have concentrated on synthetic pathways to replace formaldehyde content. For instance, much better thermal stability and functional properties were achieved by incorporating urea-formaldehyde resin/reactive kaolinite composites with 20–40% kaolinite. Some of the characteristics showed improvement in water resistance, thermal stability, and self-healing properties by the incorporation of urea-formaldehyde resin/reactive montmorillonite composites^9–12^. However, they could not completely replace the use of formaldehyde. The textile sector has a significant challenge in developing ecologically acceptable procedures. The investigation of Kabir et al., 2018 showed that a satisfactory color yield was obtained by mixing jackfruit gum with an additive in a ratio of Jackfruit latex gum to the synthetic binder of 80% and 20% ^13^. Binder extracted in nano-size particles can enhance better crosslinking and adhesion qualities resulting in reduced energy consumption and short reaction time. El-Shemy et al., 2017 studied the production of nano-binders from renewable materials using a quick and efficacious microwave process with plant oils like - sunflower and soyabean oil^14^. Fluorescent pigment latex (FPL) was created by the investigation by Li et al., 2018 using mini-emulsion polymerization and then printed on cotton fabric^5^. The main drawback of the study is that the application is limited to cellulosic fabric. The stiffness in hand feel property is one of the disadvantages because of the large particle size of the binder^5,15^. Pigment printing using a hybrid binder incorporating jackfruit latex gum produced acceptable printing results which can replace the use of toxic synthetic binder^4^.

In search of binders, a novel, and eco-friendly binder must have to be taken into account with various curing times. Chitosan is a linear polysaccharide and the second most abundant biopolymer after cellulose^16,17^. The extraction method of chitosan is previously described^18^. Typically, chitosan is extracted by the chemical extraction method. Chitin is prepared by demineralization and deproteinization of the raw materials^19^. After the deacetylation process of the chitin, chitosan is expected to be extracted. The characterization of the chitosan sample will be evaluated in the study of Trung et al., 2020 ^20^. The need for environmentally friendly binders with great color fastness has grown in recent years, and several studies have concentrated on natural binders such as chitosan^13^. The study of Xu et al., 2016 showed the effectiveness of bacterial reduction rate in a combination of silver nanoparticles with chitosan extract. Although the fabrics’ Ag content dropped to 37.6% after 30 consecutive laundry cycles, they nevertheless exhibited bacterial reduction rates of above 95% against S. aureus and E. coli^21–23^.

Organic thickening agents based on polysaccharides derived from plant exudates, such as gum karaya, gum tragacanth, and gum Arabic, as well as extracts of seaweed, such as alginate, as well as the seed or root of plants, such as guar gum and locust bean, are excellent thickeners for textile printing, but they haven’t gained much traction in pigment printing because of their relatively high solids content and negative impact on the feel^1,24^. Synthetic thickeners may be replaced with eco-friendly, natural thickeners that are non-allergic to the skin, and non-toxic^25^. Moreover, thickeners made from natural plants are non-toxic, affordable, environmentally friendly, and do not make cloth rigid. The aloe vera plant is referred to as a “healing plant.” Several studies by researchers claim that using aloe vera to treat wounds can hasten wound healing and provide UV protection^26^. Aloe vera polysaccharide gel is an excellent thickening agent having great anti-oxidant and anti-microbial properties and viscosity. Different additives can be mixed with aloe vera polysaccharides (e.g. sodium alginate) to increase the rheological performance^1^. The study by Saad et al., 2021 claimed to achieve a prominent print quality at the print paste made with an aloe vera to the additive ratio of 70:30 on cotton, wool, and polyester fabrics^27^.

In the printing process, urea is commonly used as a hydrotropic agent. The key roles of urea during the implementation of reactive dyes have been shown to include dye solubility in the reaction media, dye disaggregation, retardation of moisture during drying, and swelling of cotton, hence enhancing the dye-fiber affinity^28^. The hydrotropic agent solubilizes the hydrophobic compounds (in an aqueous solution) including – Polyethylene Glycol (PEG 400) can be used instead of urea^29^. PEG-400 has the potential to have an amphiphilic character, which may imply superior hydrotropic properties as compared to urea^30^. The composition of PEG-400 to urea can be a solution to the challenge of overcoming the environmental issues caused by textile wet processing^29^. PEG-400 can withstand aggregation of the biomaterials and plays a vital role in the protection of the printed surface^31^. It helps form H-bond with cellulose chains, thus enhancing cellulose swelling and improving dye penetration into cotton fibers^4,30^.

Printing is an example of a prominent industrial textile processing unit. Environmental considerations like as formaldehyde releases and carbon dioxide concentration must be considered. During the printing process, screen clogging due to formaldehyde releases must also be considered^32^. The printing process produces a large volume of sewage, which has a high biological oxygen demand (BOD) and chemical oxygen demand (COD). As a result, print-paste composition and printing procedures should be reconsidered to fulfill environmental challenges.

Cellulosic materials have been acknowledged as suitable substrates for the development of microorganisms, such as bacteria, fungi, etc. Which results in irritation, unpleasant odor, decoloration, stains, partial deterioration, and a higher chance of contamination^3^.

## Materials and experimental

### Materials

Cotton (100%), poly-cotton (polyester: cotton = 65:35), and Linen (100%) fabrics were used. The fabrics were sourced from Pakiza Knit Composite Limited and BEXIMCO Textiles & Apparel Division, BEXIMCO Limited. The natural gel was extracted from the leaves of aloe vera plants to use as an eco-friendly thickener.

The Chitosan was extracted from the shrimp shell to use as an eco-friendly binder. The shrimp shells were obtained from Karwan Bazar, the largest commodity marketplace in Dhaka, Bangladesh.

Sodium Hydroxide, Sodium Alginate, Pigment, Polyethylene Glycol-400 (PEG-400), Acetic acid, and other required chemicals were purchased from Tikatuli, Dhaka, Bangladesh.

#### Extraction of chitosan

##### Pretreatment of shrimp shells

Chitosan was used in this project to act as a binder. The extraction procedure of Chitosan is significant as a successful extraction may raise the quality and durability of the print paste. The shrimp shells were first shredded into small pieces by using a blender so that they could cover more surface area during the chemical treatment. Afterward, shrimp shells having 80% moisture content were pretreated with warm water several times. After the rinsing process and being dried, the shrimp shells were treated in 0.4 M HCl solution. After the pretreatment, the samples were dried before subsequent processing^17,18,24^.

##### Preparation of chitin

In this research, we utilized the chemical treatment process to synthesize chitosan^19,33^. The pretreated samples were demineralized with 1 M HCl solution in the ultrasonication method for 24 h at room temperature (± 30 °C) at material and liquor ratio (M: L) of 1:10 ^34,35,^. The mineral matter from the shrimp shells was removed in this process. The demineralized samples were treated in an ultrasonicator for deproteinization by treating with 0.8 M NaOH solution at room temperature (28–32 °C) for 24 h in a material and liquor ratio (M: L) of 1:10 ^20^. The ultrasonicator was equipped with a sonication frequency of 37 kHz for all the processes. The resultant chitin is dried in sunlight and stored^16,23,26,36^.

##### Preparation of chitosan

**Fig. 1:**
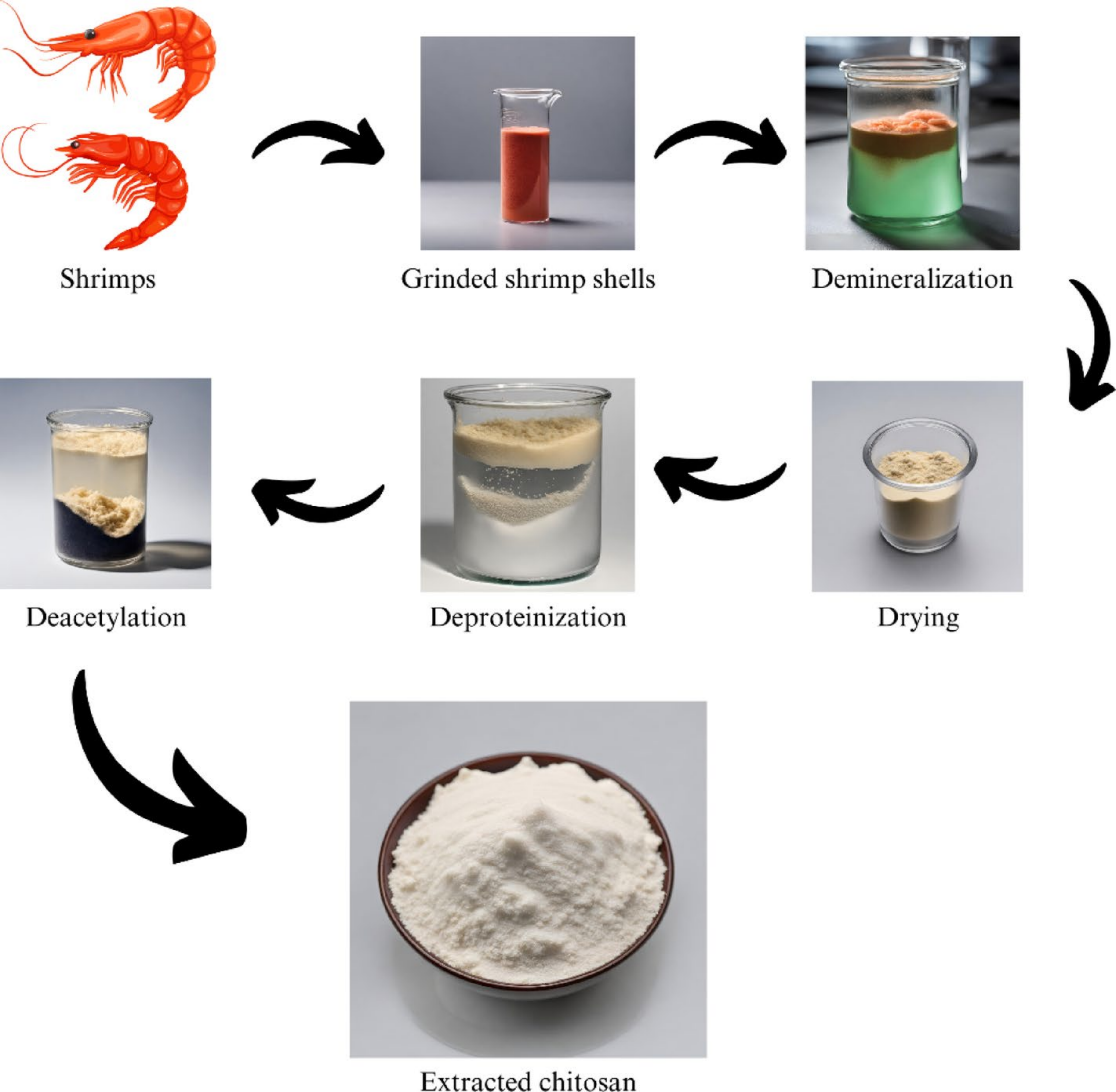
Chitosan extraction.

Chitosan was prepared using the method described previously^20^. Typically, the extracted chitin was Deacetylated with a 50% NaOH solution. The chitin was treated with 12.5 M NaOH solution and incubated at 65 °C for 12 h. This process was carried out two times after drying the specific batch of units to obtain more accuracy in extraction by reducing the contaminants. By the deacetylation process, the acetyl groups of the chitin samples were removed, and the resultant Chitosan was extracted^5,14^. Figure 1 shows the chitosan extraction steps.

##### Preparation of chitosan gel

**Fig. 2:**
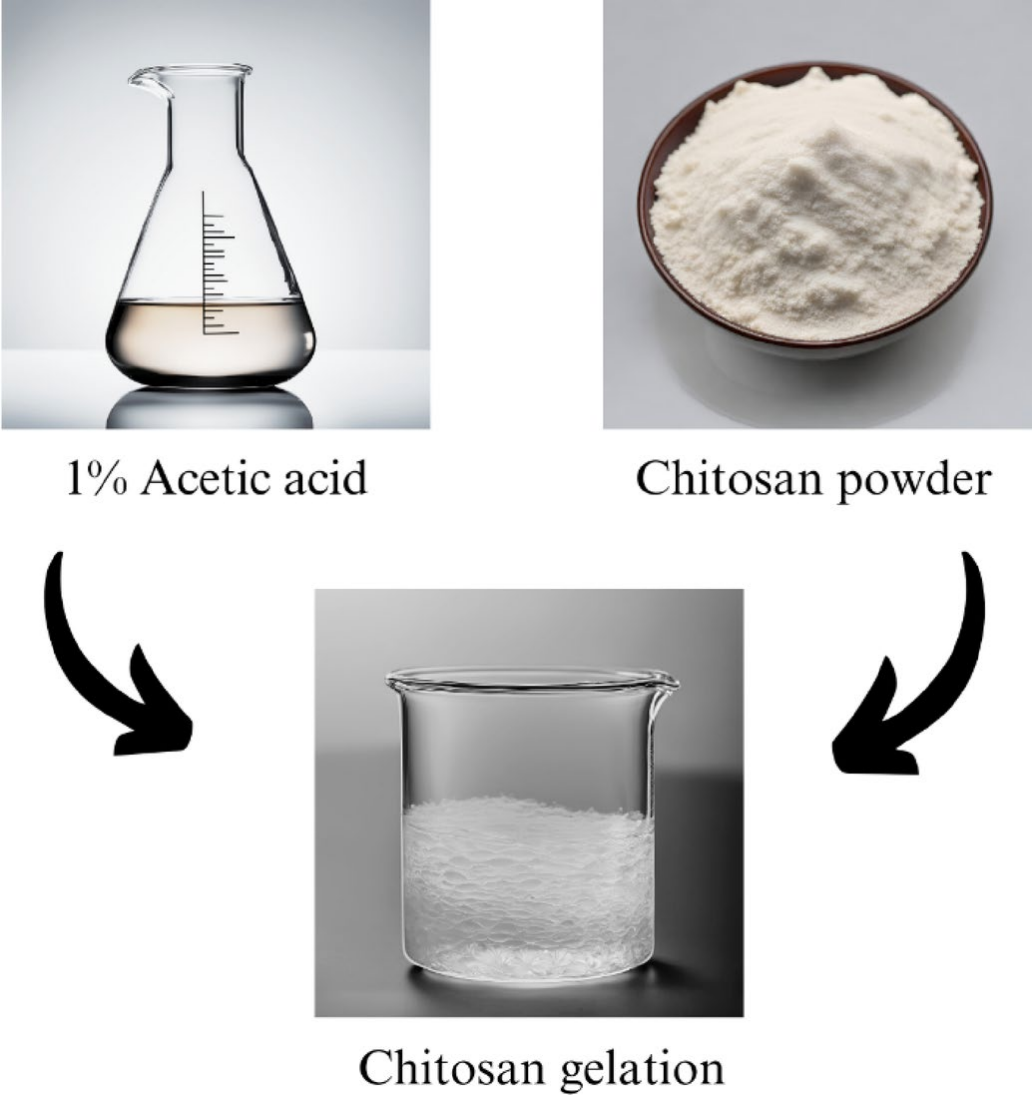
Chitosan gel preparation.

Chitosan gel was prepared by preparing a stock solution. A solution of 1% CH_3_COOH was prepared before the process. The pH level of the solution should be 4 in this case^20,37^. After that, freshly extracted chitosan powder of 3% w/v was added to the acetic acid solution and stirred thoroughly till its gelation^34,38^. The chitosan gelatin was stored in a refrigerator at 4 °C to keep it free from mold and any unwanted bacterial attack^19^. Figure 2 shows the gel preparation from chitosan.

#### Extraction of aloe vera gel as thickening agent

Aloe vera leaves contain greater gel contents than other portions of the plant; larger, thicker leaves were picked for gel separation. Laying the leaves flat on a cutting surface, approximately 1.5 inches of their tips were sliced off using a knife. Both long edges of the leaves were removed^27^. The leaves were sliced into the upper and lower half from top to bottom. The two parts were placed face up and side by side. A fork was used to extract the gel from the leaves. The gel was put in a jar and kept in the refrigerator and away from sunlight to prevent spoiling hence fungal attack and their rheological behavior^39–41^.

### Experimental

#### Preparation of screen

The photochemical method is most widely used for preparing the screen. This is based on the principle that when a coating of a solution of ammonium dichromate-gelatine or ammonium dichromate-polyvinyl alcohol is dried and exposed to light^42^.

#### Preparation of print paste

**Fig. 3:**
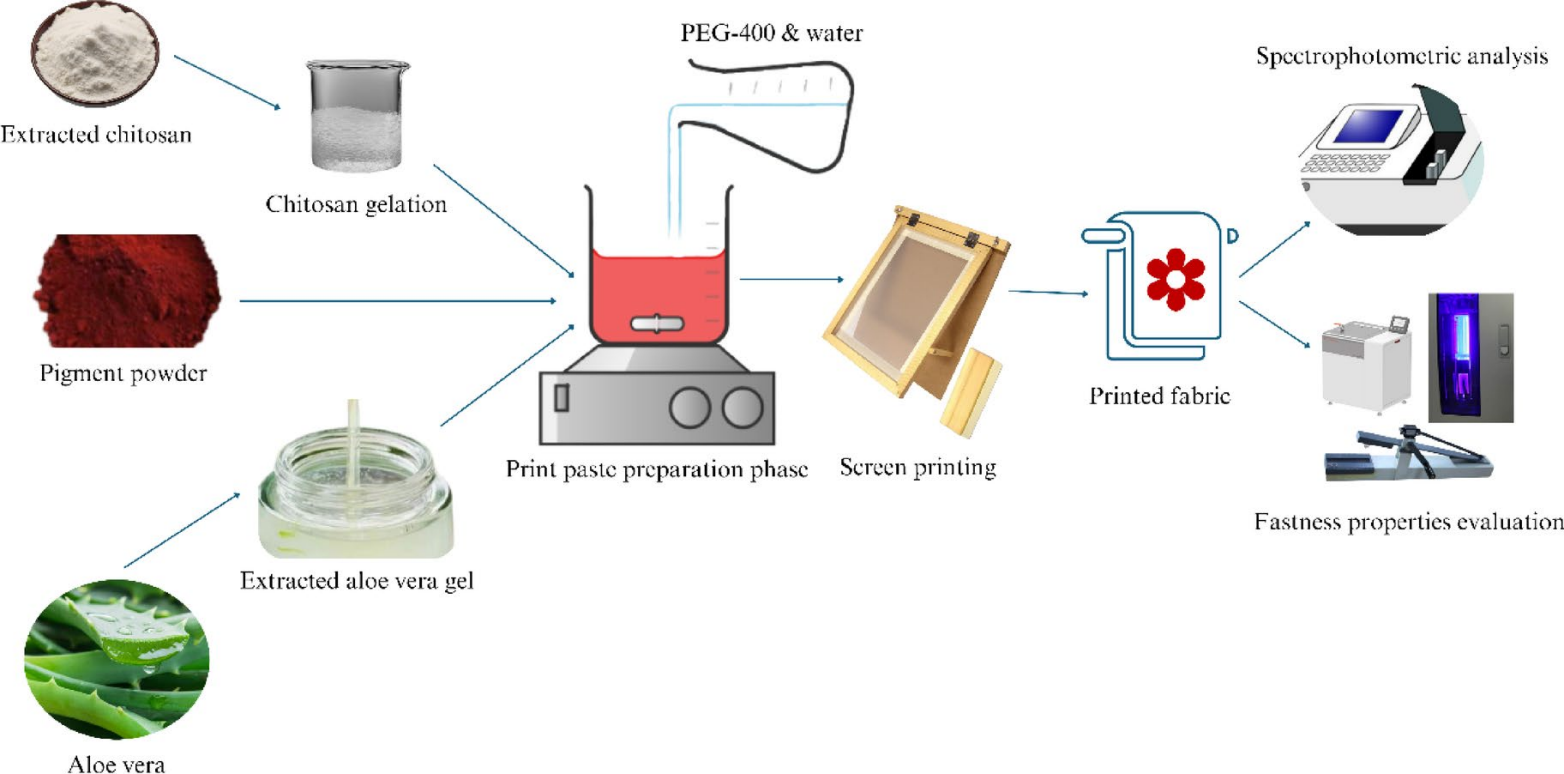
Schematic Diagram of Sustainable Printing Process.

Two distinct print pastes were prepared for the test to examine their post-printing performance. Figure 3 depicts the schematic representation of the process flow for the proposed sustainable printing. Chitosan was used as the binder in place of Zytrol 700 (emulsion), plant-derived aloe vera gel was used as the thickener in place of sodium alginate, and PEG-400 was used as the fixer in place of urea. The recipe of the pastes for both conventional and sustainable printing is shown in Table 1.

**Table 1:** Recipe of the pigment printing paste for 100 mL printing paste.

Conventional Printing ingredients	Sustainable Printing ingredients
Zytrol 700 (emulsion) = 10gm	Chitosan = 10gm
Pigment = 5gm	Pigment = 5gm
Sodium Alginate = 8gm	Aloe Vera = 8gm
Urea formaldehyde = 1gm	PEG-400 = 1gm
Water = 76 ml	Water = 76 ml

#### Color fastness

Color fastness is an important assessment by which, we can declare the stability and strength of the color imparted according to a recognized standard. Typically, color bleeding and staining are assessed through this process. Color fastness to wash, light, and rubbing was carried out according to the test standard ISO 105-C06. Typically, fabric samples were treated with ECE detergent for 30 min at 40 °C^43,44^.

##### Color fastness to wash

The ISO 105-C06 standards were maintained while conducting the wash fastness test for the printed sample. Typically, the printed fabric samples having the dimension of (15 × 4) centimeters were cut and sewn along with a multifiber fabric having the same dimension. The cut fabric samples were treated in a solution of standard detergent (ECE) at 40 °C for 30 min^1,45^. The same process was carried out for all the samples individually. After the wash treatment, samples were dried before the after-wash result inspection.

##### Color fastness to light

A blue wool scale ranging from one to eight was used in this instance of light fastness. One indicates a poor fastness property, and eight indicates the best fastness property to the color fastness to light. The “ISO 105-B01 Grade 4” criterion was perpetuated for the light fastness assessment^46^. Specimen were cut into (15 × 4) centimeters. electrically powered xenon arc fading illumination was employed throughout the test^47^. Consistent lab conditions of temperature and humidity were maintained and it was: 25 °C and 65% ^48^.

##### Color fastness to rubbing

The rubbing fastness test is a significant characterization that ensures the printed substance from prolonged exposure to roughness and its ability to withstand harsh environments due to wear and tear. Several factors are the key parameters of the rubbing fastness test of materials, such as wettability of materials, hydrophilicity, temperature, and dry and wet conditions; as in this research, we induced biomaterials in the printing paste. An electric crock meter was used for occupying the test. Standard lab conditions were maintained, which is following ISO 105-X12:2001 were used to test the rubbing fastness test^49,50^. Test samples were cut into (20 × 5) centimeters in dimension and put on the crock meter’s panel^51^. Using a scraping substance, ten strokes were made in both the dry and wet conditions^27^.

#### Spectrophotometer test

Plenty of data on the printed sample can be found by the spectrophotometer test. In this process, the “Spectra flash Datacolor SF 600 (USA)” was used. For achieving more precise results of the test, the “GretagMacbeth SpectroScan” was used to examine the reflectance and the spectrum of the color produced. The CIELAB color coefficients (CIE L*, a*, b*, C*, h*) and the color strength (K/S) in the visible spectrum (400–700 nm) were assessed using the Kubelka-Munk equation^52–56^

1 $$\:\frac{K}{S}=\frac{(1-{R)}^{2}}{2R}$$

Here, K represents the absorption coefficient, R is the reflectance of the dyed sample, and S signifies the scattering coefficient.

2 $$\:{\varDelta\:E}_{ab}^{*}=\sqrt{{(\varDelta\:L)}^{2}+{(\varDelta\:a)}^{2}+{(\varDelta\:b)}^{2}}$$

Typically, ∆E* is the overall discrepancy on the CIELAB diagram. ∆L depicts the variation in lightness/darkness value, ∆a denotes the variation along the red/green axis, and ∆b indicates the variation along the yellow/blue axis^56^.

## Results and discussion

### Color fastness

The color fastness tests were occupied along with the color staining to wash. Color fastness to rubbing was examined in both dry and wet conditions. Optimum lab conditions were strictly maintained during the tests. Color fastness and color staining results of conventional and sustainable printing for the non-washed and 10 times washed specimens are depicted in Table 2.

**Table 2:** Color change and color staining results for non-washed and after 10-washed specimens.

Sample	Color change				Color staining to wash (Multifiber fabric rating)					
	Wash	Light	Rubbing		Wool	Acrylic	Polyester	Nylon	Cotton	Acetate
			Dry	Wet						
Cotton (Conventional)	4/5	7	3/4	2/3	5	4/5	5	5	5	5
Poly-cotton (Conventional)	5	7/8	4	3	5	5	5	4/5	5	5
Linen (Conventional)	5	7/8	4	3	4/5	5	4/5	5	5	4/5
Cotton (Sustainable)	3/4	6/7	3/4	2	3/4	4	4	4	3/4	4
Poly-cotton (Sustainable)	4/5	6/7	3	2/3	3/4	4/5	4/5	4	4	4/5
Linen (Sustainable)	4	7	3/4	2/3	4	4/5	4/5	4	4/5	4/5
10 times washed Cotton (Conventional)	4/5	7/8	3/4	3	4/5	5	5	5	4/5	5
10 times washed Poly-cotton (Conventional)	5	7/8	4	3	5	5	4/5	5	4/5	5
10 times washed Linen (Conventional)	5	7/8	4	2/3	4/5	5	5	5	4/5	5
10 times washed Cotton (Sustainable)	4	6/7	3	2/3	4/5	4/5	5	5	4	4/5
10 times washed Poly-cotton (Sustainable)	4	7/8	3/4	3	4/5	5	5	5	4	5
10 times washed Linen (Sustainable)	4/5	7	3/4	3	4/5	5	5	5	4/5	5

With a grayscale rating ranging from 4/5 to 5, conventional printing shows very good to excellent wash fastness, suggesting that there is little color change after washing. Strong resistance to fading is shown by the wash fastness test, which stays consistent even after 10 washes. In contrast, sustainable printing begins with moderate to good wash fastness (3/4 to 4), which means that color change is initially a little greater. Nevertheless, its performance rises to a 4 to 4/5 after 10 washes, exhibiting improved color retention over time, particularly on linen.

The light fastness results of the conventional prints keep excellent light fastness, with blue-wool scale ratings of 7 to 7/8, showing strong resistance to color fading under light exposure. These results stay unchanged even after 10 times washed sample. Sustainable prints initially show moderate to good resistance to light exposure (6/7 to 7), meaning they are slightly more prone to fading under prolonged exposure to light. However, in poly-cotton, the light-fastness improves to 7/8 after 10 times washing, accomplishing the conventional printing rating. This suggests that the color stability of sustainable prints improves after 10 times washed samples.

The dry rubbing fastness in terms of traditional printing performs relatively well in dry rubbing resistance, with scores of 3/4 to 4 (moderate to good), meaning it can endure friction without substantial color loss. Sustainable printing has slightly lower resistance, with a rating between 3 and 3/4 (average to moderate), indicating it is more prone to minor surface wear but still holds up well. Both printing methods demonstrate weak resistance to wet rubbing, with conventional printing scoring 2/3 to 3 (poor to average) and sustainable printing ranging from 2 to 3 (poor to average). This shows that both techniques struggle with conserving color shades when exposed to friction in humid environments.

The color staining test observations demonstrate that traditional printing achieves very good to exceptional results, with staining ratings of 4/5 to 5, suggesting negligible color transfer into adjacent materials while washing. Sustainable printing initially displays moderate to very good performance (3/4 to 4/5) but improves after 10 times washing, reaching 4 to 5, which is comparable to conventional prints with the same condition. This implies that while sustainable prints may transfer some color initially, their durability increases in the 10 times washed specimen.

Information stated in Table 2 clarified that the printing specimens with the conventional method showed prominent characteristics in color fastness and color staining compared to sustainable printed specimens, whereas the specimens with sustainable printing also challenged the conventional method in significant aspects. In contrast to its predominance in environmental aspects, sustainable printing also demonstrated superior results in the color-staining on multifiber fabric. Our study found that the washed specimens had greater color fastness and color staining. This phenomenon was inspected because there were some temporary or protruding auxiliaries present on the fabric samples which were washed out after washing the samples hence increasing the cohesiveness of the bonded print paste onto the fabric.

### Spectrophotometric test

**Fig. 4:**
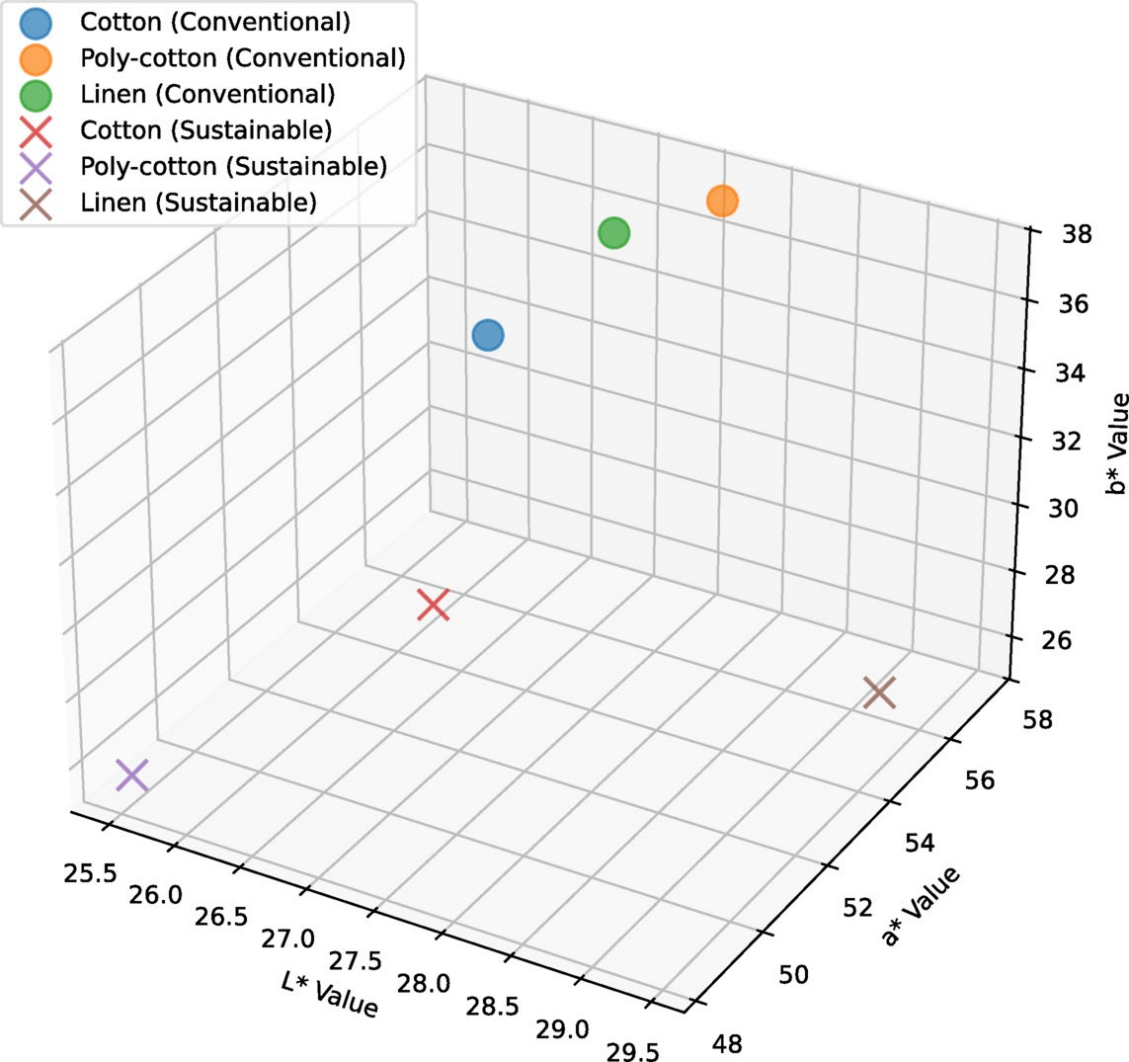
3D scatter plot of CIE L*a*b* values for non-washed specimens.

The test was occupied individually for both the unwashed and 10 times washed printed specimens. For more accuracy of the data, the test was done several times keeping the samples in the same condition prior to analysis for further justification. An average of 5 readings were analyzed for precision. CIE L*a*b* Explanation: L*: Indicates the degree of brightness, with 0 denoting black and 100 denoting white, a*: Denotes the range of value between green (negative values) and red (positive values), b*: represents the region that lies between blue (negative values) and yellow (positive values). Figure 4 shows the CIE L*a*b* coordinates for the non-washed specimens. Here, L*a*b* values were plotted in 3-dimensional x, y, and z coordinates respectively.

From the figure, the conventional cotton fabric showed CIE L*a*b* values of 26.56, 54.61, and 34.37 respectively. For conventional poly-cotton, the values are: 27.64, 57.38, and 37.22 which is slightly more than that of the conventional cotton fabric in all three coordinates. Conventional linen showed 27.05, 56.46, and 36.40 respectively, which is slightly higher than that of conventional cotton but lower than conventional poly-cotton in all three coordinates. Similarly, the sustainable cotton showed CIE L*a*b* color coordination values of 26.96, 51.46, and 29.56. Sustainable poly-cotton showed 25.50, 48.33, and 25.57, which is slightly less than that of sustainable cotton fabric in all three coordinates. The values of sustainable Linen are: 29.44, 54.87, and 26.83, which is higher than that of sustainable cotton and sustainable poly-cotton in L* and a* values but lower than sustainable cotton in b* value.

**Fig. 5:**
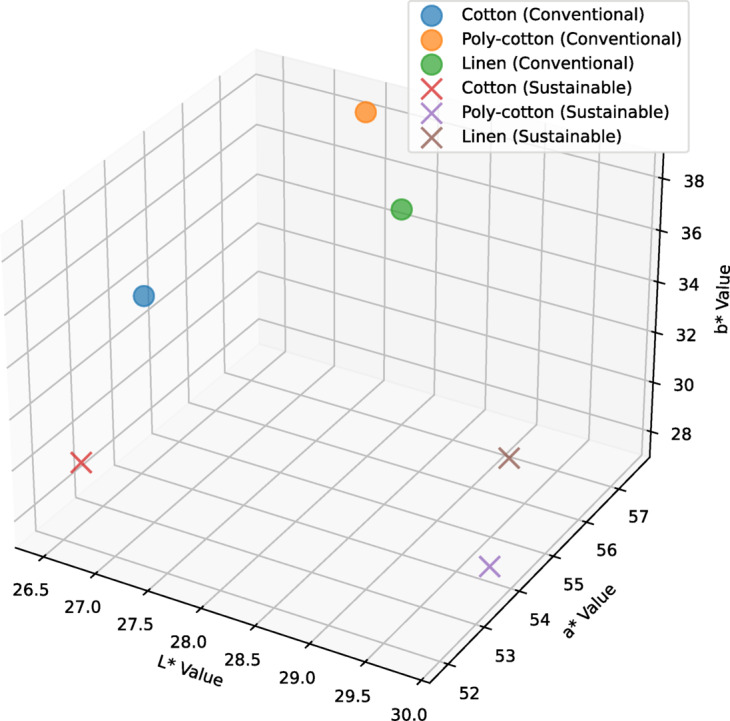
3D scatter plot of CIE L*a*b* values for washed specimens.

Figure 5 shows the CIE L*a*b* coordinates for the washed specimens.

From the above illustration, the CIE L*a*b* values of conventional cotton are: 26.47, 54.25, and 33.72 respectively. Conventional poly-cotton showed 27.45, 57.57, and 38.2 which is slightly higher than that of conventional cotton in all three coordinates. The conventional linen showed 27.89, 57.27, and 35.21 which is higher than conventional cotton but lower than conventional poly-cotton in a* and b* values. For sustainable cotton, the values are found 26.74, 51.91, and 30.45 respectively. Sustainable poly-cotton showed a value of 29.83, 53.92, and 27.71 which is slightly better than sustainable cotton in L* and a* values but lower in b* value. Lastly, the sustainable linen showed 29.43, 55.74, and 29.03 which is slightly better than sustainable cotton in L* and a* values but lower in b* values and slightly better than sustainable poly-cotton in a* and b* values but lower in L* value.

Promising characteristics are observed in non-washed sustainable linen fabric in L*, which is even greater than the conventional printed fabrics. Similarly, the 10-washed sustainable poly-cotton and linen showed greatness in L* values. Having lower in a*, and b* increases their potentiality to have better fastness properties and color staining^48,57^. The difference between the conventional and sustainable specimens in terms of L*, a*, and b* values is not significantly notable.

## Conclusions

The traditional printing method has been in use and popular in textile industries for a long time, because of its easy processability and cost-effectiveness. However, their impact on the environment has been a major concern as the release of these harmful chemicals during screen and textile washing (after use) may cause a severe disbalance in the ecosystem. Therefore, this conventional printing does not fulfill the promise of making a sustainable future and needs an alternative eco-friendly method to print the textiles. This study successfully demonstrated a possible way to replace toxic chemicals by learning from nature such as utilizing abundant biomaterials chitosan as a binder, and aloe vera as a thickener. This study also showed these biomaterials are compatible with each other and they can be incorporated into the textiles without sacrificing the quality and performance and quality. However, challenges remain to make them industrially feasible due to the difficulty in processing and extraction of these materials from nature. This leads to an increase in overall cost which is a concern in terms of scalability and profitability. However, with further research, it will be possible to find a more sustainable textile printing method that not only ensures environmental sustainability but also economic feasibility.

## Electronic supplementary material

Below is the link to the electronic supplementary material.

Supplementary Material 1

## Data Availability

Data are provided within the manuscript and supplementary information files.
